# Dose Reduction and Diagnostic Performance of Tin Filter–Based Spectral Shaping CT in Patients with Colorectal Cancer

**DOI:** 10.3390/tomography8020088

**Published:** 2022-04-08

**Authors:** Koichiro Kimura, Tomoyuki Fujioka, Mio Mori, Takuya Adachi, Takumi Hiraishi, Hiroto Hada, Toshiaki Ishikawa, Ukihide Tateishi

**Affiliations:** 1 Department of Diagnostic Radiology, Tokyo Medical and Dental University, 1-5-45 Yushima, Bunkyo-Ku, Tokyo 113-8510, Japan; kmrdrnm@tmd.ac.jp (K.K.); mrdrnm@tmd.ac.jp (M.M.); adcdrnm@tmd.ac.jp (T.A.); ttisdrnm@tmd.ac.jp (U.T.); 2 Department of Radiology, Tokyo Medical and Dental University, 1-5-45 Yushima, Bunkyo-Ku, Tokyo 113-8510, Japan; hriscrad@tmd.ac.jp (T.H.); hdcrad@tmd.ac.jp (H.H.); 3 Department of Specialized Surgeries, Tokyo Medical and Dental University, 1-5-45 Yushima, Bunkyo-Ku, Tokyo 113-8510, Japan; ishi.srg2@tmd.ac.jp

**Keywords:** dose reduction, spectral shaping technique, colorectal cancer

## Abstract

Routine CT examinations are crucial in colorectal cancer patients (CCPs); however, the high frequency of radiation exposure is a significant concern. This study investigated the radiation dose, image quality, and diagnostic performance of tin filter-based spectral shaping chest–abdominal–pelvic (CAP) CT for CCPs. We reviewed 44 CCPs who underwent single-phase enhanced tin-filtered 100 kV (TF100kV) and standard 120 kV (ST120kV) CAP CT on separate days. Radiation metrics including the volume CT dose index (CTDI_vol_), dose-length product (DLP), and effective dose (ED) were calculated for both protocols. Two radiologists assessed the presence of the following lesions: lung metastasis, liver metastasis, lymph node metastasis, peritoneal dissemination, and bone metastasis. The area under the receiver operating characteristic curve (AUC) was calculated for the diagnostic performance of each protocol. Radiation metrics of the TF100kV protocol were significantly lower than those of the ST120kV protocol (CDTI_vol_ 1.60 ± 0.31 mGy vs. 14.4 ± 2.50, *p* < 0.0001; DLP 107.1 (95.9–125.5) mGy·cm vs. 996.7 (886.2–1144.3), *p* < 0.0001; ED 1.93 (1.73–2.26) mSv vs. 17.9 (16.0–20.6), *p* < 0.0001, respectively). TF100kV protocol achieved comparable diagnostic performance to that of the ST120kV protocol (AUC for lung metastasis: 1.00 vs. 0.94; liver metastasis: 0.88 vs. 0.83, respectively). TF100kV protocol could substantially reduce the radiation dose by 89% compared to that with the ST120kV protocol while maintaining good diagnostic performance in CCPs.

## 1. Introduction

Colorectal cancer is the third most commonly occurring malignancy in the world [1]. Annually, there are over 1.9 million newly diagnosed colorectal cancer patients (CCPs) and 900,000 colorectal cancer-related deaths [1,2]. Even after curative treatment, tumors recur in 30% of CCPs with stage I–III and 65% of CCPs with stage IV [3,4,5,6,7,8,9,10,11]. However, prognoses of CCPs have improved over the past three decades as a result of the development of multimodal treatments [12,13].

The chest–abdominal–pelvic (CAP) computed tomography (CT) scan is the standard imaging method used for monitoring CCPs during follow-up [14]. Most surveillance guidelines recommend follow-up CT scans to be performed every 3–12 months for 2–3 years following curative treatment, with a decreased frequency of assessments at 3–5 years post-treatment [15,16,17,18,19]. CT is also used to evaluate response to chemotherapy, and in clinical practice, it is commonly employed for CCPs with unresectable and metastatic tumors every 2–3 months based on clinical trial designs [20,21]. Because of such a routine procedure, radiation exposure due to routine CT examinations during postoperative follow-up and chemotherapy is higher in CCPs, which has become a significant concern. Thus, reasonable dose reduction while ensuring adequate diagnostic performance is needed for CT examination of CCPs during postoperative follow-up or chemotherapy.

Based on the principle of as low as reasonably achievable (ALARA), considerable effort has been made toward reducing the radiation dose of CT devices. The spectral shaping technique, which involves adding a tin filter (TF) on the scanner x-ray tube, is a powerful method for reducing radiation dose. TF minimizes the bulk of lower-energy photons, which are less relevant to image quality but increase the radiation exposure of patients [22,23]. In addition, TF reduces the effect of beam hardening artifacts, which improves the quality of reconstructed CT images. Previous studies have shown an advantage of the TF protocol over standard protocols for image quality and radiation dose reduction for chest, abdominal, paranasal sinus, and pediatric CT [22,23,24,25,26,27]. However, the utility of TF-based CAP CT in CCPs during postoperative follow-up and chemotherapy compared with the standard protocol CAP CT has not been comprehensively examined. Therefore, this study investigated radiation exposure, image quality, and diagnostic performance of TF-based spectral shaping CAP CT in CCPs compared to the standard protocols.

## 2. Materials and Methods

### 2.1. Patient Data

**Figure 1:**
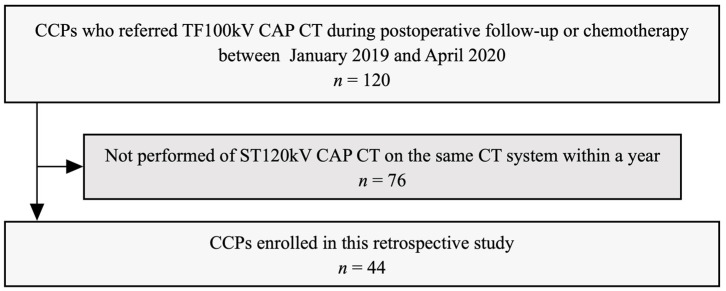
Recruitment pathway for patients in the present study CCPs, colorectal cancer patients; TF100kV, tin-filtered 100 kV; CAP, chest–abdominal–pelvic; ST120kV, standard 120 kV.

This retrospective study was approved by the local institutional review board (M2020-071), which waived the requirement for informed consent. We identified the 120 CCPs who were referred for TF100kV CAP CT during postoperative follow-up or chemotherapy from our radiologic databases between January 2019 and April 2020. The patient selection process of the study is shown in Figure 1. The inclusion criteria were imaging the standard 120 kV (ST120kV) CAP CT on the same CT system within one year of undergoing TF100kV CAP CT. There were no exclusion criteria. Thus, a total of 44 CCPs were included in our study. Patient clinicopathological data were extracted from electronic medical records. The tumor-node-metastasis staging system was used according to the edition valid at the time of cancer diagnosis. A radiologist determined the presence of metastases in each patient at different time points, both the TF100kV and ST120kV protocol scans, respectively, based on clinicopathological data as of April 2021 from electronic medical records. The metastases evaluated were as follows: (1) lung metastasis, (2) liver metastasis, (3) lymph node metastasis, (4) peritoneal dissemination, and (5) bone metastasis. The diagnostic criteria for metastasis were based on the pathological diagnosis if the biopsy was performed, or if not, clinical diagnosis using radiology reports and clinical records. We defined disease-free as patients with no evidence of new lesions on the CT images, and the tumor markers were below cut-off levels. For patients during chemotherapy, we determined that the negative of metastasis was if the target lesions completely disappeared on the CT images.

### 2.2. CT Examination Technique and Reconstruction

A single-source CT system (Siemens SOMATOM Edge Plus, Siemens Healthineers, Erlangen, Germany) was used for all examinations. Detailed CT acquisition parameters for both the TF100kV and ST120kV protocols are summarized in Table 1. All patients were examined using automatic tube current modulation for effective mAs (CARE Dose 4D) and without automated tube potential control (CARE kV). The beam pitch of the TF100kV protocol was set at 0.5 lower than the ST120kV protocol to maintain image quality. The scan range of the TF100kV and ST120kV protocols covered the entire CAP area, which extended from the upper level of the thyroid to the great trochanter. In all patients, a deep-inspiration breath was held during scan acquisition. A contrast medium of 300 or 350 mg/mL iodine concentrations amounting to 1.6–2.0 mL/kg of body weight was administered intravenously within 50 s using a pump injector without a saline flush.

By default, both the TF100kV and ST120kV images were reconstructed to a slice thickness of both 2.0 mm and 5.0 mm, respectively, with no interslice gap, using a soft tissue kernel (Bf37) for the parenchymal analysis and a sharp tissue kernel (Bl57) for the lung analysis. The TF100kV and ST120kV protocols for the parenchymal images were reconstructed using the iterative reconstruction (IR) technique (ADMIRE: Advanced Modeled Iterative Reconstruction) set at strength levels of 4 and 2, respectively. Additionally, the lung images of both protocols were reconstructed using the filtered back-projection technique.

### 2.3. Radiation Metrics of ST120kV and TF100kV Protocol

To estimate radiation doses, we recorded the dose parameters for each patient and each acquisition protocol. Volume CT dose index (CTDI_vol_), dose-length product (DLP), and effective tube current (mAs) were extracted from the dose report and DICOM data. The effective dose was estimated by multiplying the DLP by the standard conversion factor for adult abdominopelvic CT of 0.018 mSv mGy^−1^ cm^−1^ [28]. Size-specific dose estimates (SSDE) were calculated using appropriate conversion factors based on the use of the 32-cm phantom for CTDIvol based on the following equation: SSDE = *f*_size_ × CTDI_vol_ [29].

### 2.4. Assessment of Objective Image Quality

For the quantitative evaluation, we measured Hounsfield unit (HU) values and image noise (standard deviation [SD] of measured HU values) by placing 1.1 cm^2^ oval regions of interest (ROIs) within the liver, abdominal aorta, and spinal erector muscles on the reconstructed 2 mm axial CT images of both protocols. Image noise was determined as the SD of an ROI placed in the air space outside the anterior abdominal wall. Each ROI was drawn on an identical or nearly identical segment using a picture archiving and communication system (Rapideye, Canon, Tokyo, Japan) by the same radiologist with seven years of CT imaging experience. Based on these measurements, contrast to noise ratio (CNR) and figure of merit (FOM) were calculated for each image dataset using the following equations: CNR = (mean HU of area of interest − mean HU of the erector muscle)/(SD of the erector muscle); and FOM = CNR^2^/effective dose.

### 2.5. Assessment of Diagnostic Performance for Metastases

Two additional radiologists (with eight and 12 years of experience in CT imaging, respectively) who were blind to the acquisition protocol and patient clinical data (including imaging reports and prior examinations) independently assessed the presence of the listed metastases. They evaluated the images of each protocol in random order using a 5-point scale (1, no findings of metastasis; 2, low probability of metastasis; 3, intermediate; 4, probable presence of metastasis; and 5, presence of metastasis). A score of ≥4 was defined as the presence of metastasis, whereas a finding of ≤3 was considered as the absence of metastasis. Discrepancies were resolved two weeks after the independent assessments by consensus.

### 2.6. Assessment of Subjective Quality of TF100kV Images

Two radiologists independently evaluated the subjective quality of TF100kV images, the purpose of which was to detect the listed metastases. We used a 5-point scale for the reference (1: very poor, 2: poor, 3: moderate, 4: good, and 5: excellent). A score of ≥3 was defined as acceptable for use in clinical practice, whereas a score of ≤2 was considered unacceptable.

### 2.7. Statistical Analysis

All statistical analyses were performed using the JMP 14.2 statistical software (SAS Institute Inc., Cary, NC, USA). Parametric data were displayed as means ± SDs and were tested and compared using Paired *t*-tests, whereas non-parametric data were reported as median (interquartile range [IQR]) and compared using the Wilcoxon signed-rank test. Kappa statistics were used to assess interreader agreement of diagnostic performance. Kappa (k) values were calculated separately for each metastasis and were interpreted as follows: <0.20, slight agreement; 0.21–0.40, fair agreement; 0.41–0.60, moderate agreement; 0.61–0.80, substantial agreement; and 0.81–1.00, almost perfect agreement. Receiver operating characteristic curve analyses were performed, and area under the curve (AUC) values were calculated to assess the diagnostic performance for metastasis of each protocol and were interpreted as follows: 0.51–0.60, fail; 0.61–0.70, poor; 0.71–0.80, fair; 0.81–0.90, good; and 0.91–1.00, excellent. We calculated the sensitivity, specificity, and accuracy for differentiating the presence or absence of the listed metastases in both protocols. A *p* < 0.05 was considered statistically significant.

## 3. Results

### 3.1. Patient Characteristics

The baseline characteristics of all patients are listed in Table 2. Mean age of patients was 65.1 years. Forty-four eligible patients had 45 colorectal cancers that were composed of three tumors in the cecum/appendix, four in the ascending colon, three in the transverse colon, one in the descending colon, 12 in the sigmoid colon, and 22 in the rectum. One patient had two separate synchronous cancers (in the transverse and sigmoid colon). Ten patients (23%) were diagnosed as Stages Ⅰ and Ⅱ, and the remaining 34 (77%) were diagnosed as Stages Ⅲ and Ⅳ.

Twenty-six patients who were disease-free and during follow-up had no evidence of new metastasis until the end of the study.

Eighteen patients were during chemotherapy and had 32 sites of metastases as of the ST120kV scans. One patient with liver metastasis and two patients with peritoneal dissemination were pathologically confirmed metastases. Of the remaining 29 sites, four sites (three patients with liver metastasis and one patient with lung metastasis) were recurrences of the residual organ after the treatment of the metastatic resection. The remaining 25 sites were diagnosed as metastases by the clinical course.

One patient with liver metastasis and one with lymph node metastasis showed the disappearance of the lesions on TF100kV images. These lesions showed no signs of re-progression until the end of the study. Therefore, they were determined to be negative for metastasis at the TF100kV scan.

None of the patients showed new site lesions during chemotherapy between the ST120kV scan and the TF100kV scan. On the other hand, new site lesions and metastases that could not be identified on the TF100kV images appeared in four patients after the scan; two patients with lung metastasis, one with lymph node metastasis, and one with peritoneal dissemination.

### 3.2. Radiation Dose

**Figure 2:**
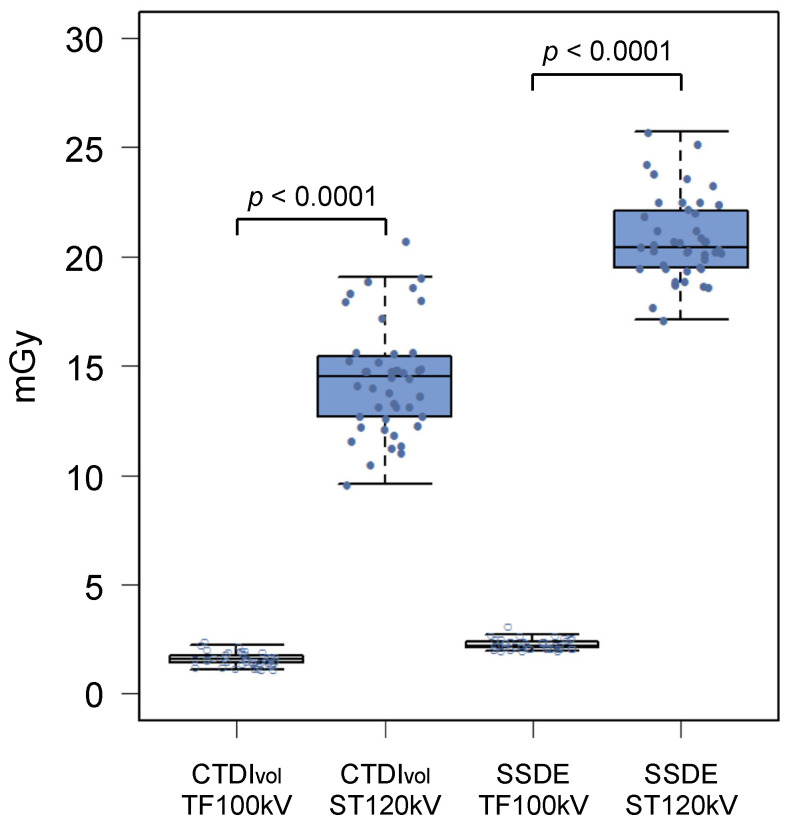
Box and whisker diagrams of CTDIvol and SSDE for the tin-filtered 100 kV (TF100kV) and standard 120 kV (ST120kV) protocols.

Radiation metrics are summarized in Table 3. The mean CTDIvol was 1.60 ± 0.31 mGy for the TF100kV protocol and 14.4 ± 2.50 mGy for the ST120kV protocol, which differed significantly (*p* < 0.0001; Figure 2). Mean DLP of the TF100kV protocol was significantly lower than that of the ST120kV protocol (107.1 (95.9–125.5) mGy cm vs. 996.7 (886.2–1144.3) mGy cm, *p* < 0.0001). The mean effective dose was 1.93 (1.73–2.26) mSv for the TF100kV protocol and 17.9 (16.0–20.6) mSv for the ST120kV protocol (*p* < 0.0001), which corresponded to an 89.2% lower dose using the TF100kV protocol compared with the ST120kV protocol. The effective dose calculated for the TF100kV protocol was close to 2 mSv. In addition, the TF100kV protocol had a significantly lower mean SSDE than the ST120kV protocol (2.29 ± 0.24 mGy vs. 20.8 ± 1.90 mGy, *p* < 0.0001; Figure 2).

### 3.3. Objective Image Quality Assessment

**Figure 3:**
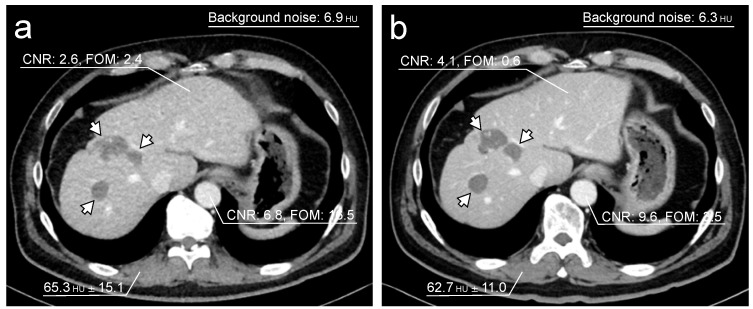
Representative single-phase enhanced CT images. Both images were acquired on separate occasions from a 62-year-old male who had liver metastases after colon cancer surgery. The tin-filtered 100 kV (TF100kV) protocol (**a**) shows lower CNR and higher FOM than the standard 120 kV (ST120kV) protocol (**b**). Liver metastases are detectable in both protocols (arrows).

The data for the objective image quality assessment are shown in Table 4. The mean background noise was significantly higher for the TF100kV protocol than for the ST120kV protocol (8.36 (7.61–8.86) HU vs. 6.04 (5.45–6.93) HU, *p* < 0.0001). CNRs of the liver and abdominal aorta were significantly higher for the ST120kV protocol than for the TF100kV protocol (liver: 2.76 (1.38–4.03) vs. 2.13 (1.42–2.73), *p* < 0.0001; abdominal aorta: 5.81 (4.76–7.52) vs. 4.08 (3.39–5.02), *p* < 0.0001). However, FOM was found to be significantly higher for the TF100kV protocol than for the ST120kV protocol (liver: 2.24 (0.89–4.07) vs. 0.36 (0.09–0.93), *p* < 0.0001; abdominal aorta: 8.62 (5.98–14.1) and vs. 1.81 (1.19–3.16), *p* < 0.0001). Figure 3 shows representative example images of both protocols.

### 3.4. Diagnostic Performance of Subjective Assessment

The distribution of diagnostic performance for the two readers’ consensus and interreader agreement is shown in Table 5. The diagnostic performance of the TF100kV protocol was comparable to that of the ST120kV protocol for lung metastases, liver metastases, peritoneal dissemination, and bone metastases (AUCs of the TF100kV protocol: 1.00, 0.88, 0.79, and 0.83, respectively; AUCs of the ST120kV protocol: 0.94, 0.83, 0.77, and 0.83, respectively). The diagnostic performance of the TF100kV protocol for lymph node metastases was inferior to that of the ST120kV protocol (AUC: 0.70 vs. 0.90). For the TF100kV protocol, false-negative diagnoses were detected in two liver metastasis cases, three lymph node metastasis cases, two peritoneal dissemination cases, and one bone metastasis case. For the ST120kV protocol, false-negative diagnoses occurred in one lung metastasis case, three liver metastasis cases, one lymph node metastasis case, two cases of peritoneal dissemination, and one bone metastasis case. Interreader agreement for the TF100kV protocol was fair to substantial (k = 0.29 for peritoneal dissemination; k = 0.66 for lung metastasis), whereas interreader agreement for the ST120kV protocol was slight to substantial (*k* = 0.17 for peritoneal dissemination; *k* = 0.72 for liver metastasis). For both protocols, lowest agreement was observed for diagnoses of peritoneal dissemination, and high agreement was observed for the assessment of lung and liver metastases.

### 3.5. Subjective Quality of TF100kV Images

**Figure 4:**
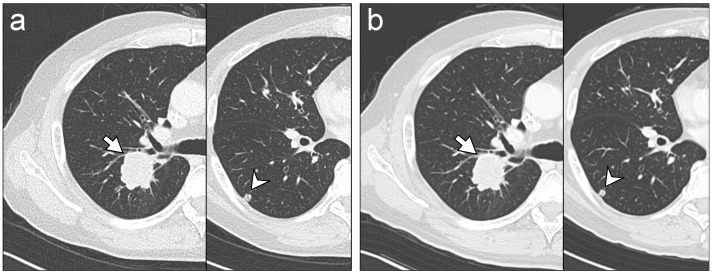
Representative chest CT images. Images were acquired on separate occasions from a 66-year-old male who received chemotherapy for lung metastases after colon cancer surgery. The readers identified lung metastasis lesions (arrows and arrowheads) on tin-filtered 100 kV (TF100kV) images (**a**) and standard 120 kV (ST120kV) images (**b**) with equal reliability, regardless.

Mean score of subjective quality of TF100kV images was >3 (moderate, TF100kV image quality is acceptable for use in clinical practice) for all lesions (Table 6). Therefore, TF100kV image quality was deemed sufficient for making diagnoses. Both readers gave a lung and bone metastases score of 4 (good diagnostic reliability) in all patients. Figure 4 shows a representative example case of lung metastasis for both protocols.

## 4. Discussion

Our study demonstrated that TF100kV CAP CT can substantially reduce the radiation dose by approximately 89% compared with the ST120kV protocol. Even though image quality metrics of the TF100kV protocol such as CNR and background noise were poorer than those of the ST120kV protocol, FOM of the TF100kV protocol was significantly higher than the ST120kV protocol. The evaluation by the readers showed that the TF100kV protocol maintained a good diagnostic performance for CCPs during postoperative follow-up and chemotherapy. Moreover, both readers evaluated that TF100kV image quality was diagnostically acceptable for use in clinical practice for confirming or excluding the recurrence of colorectal cancer.

Medical radiation is increasing annually, and it is widely accepted that medical radiation exposure is largely attributed to CT examinations [30]; however, there is currently no alternative diagnostic imaging method that allows for detailed and rapid evaluation of a patient’s body. The IR algorithm, which is now one of the most ubiquitous techniques for dose reduction while maintaining low image noise, offers only modest (approximately 25%) levels of dose reduction while maintaining diagnostic performance for imaging the abdominal area [31]. Therefore, the TF100kV protocol, which enables high dose reduction while ensuring adequate diagnostic performance, may alleviate concerns of excessive medical radiation exposure of CCPs who require frequent CT examinations for routine surveillance and response evaluation.

In our study, we showed that TF100kV CAP CT had 89% lower radiation exposure compared with the ST120kV protocol but maintained almost comparable diagnostic performance. Our findings are analogous with previous studies that compared the TF protocol with the standard CT protocol for imaging individual parts of the body. Gordic et al. [23] first suggested the feasibility of the 100 kV spectral shaping protocol for chest CT for detecting lung nodules and achieved high sensitivity and diagnostic confidence with a low effective radiation dose of 0.06 mSv. Suntharalingam et al. [32] reported that the 100 kV spectral shaping protocol for whole-body bone CT for evaluating osteolytic lesions in patients with multiple myeloma could achieve adequate image quality while reducing CTDIvol and DLP by approximately 75% more than the control group. Leyendecker et al. [25] reported that in the abdominopelvic area, the TF100kV protocol for contrast-enhanced abdominopelvic CT imaging achieved a similar diagnostic performance for detecting abdominal abnormalities as the standard CT protocol while reducing CTDIvol and SSDE by 81%. Our and previous results collectively indicate that the TF protocol has high utility for reducing radiation dose in various clinical situations.

In our study, the ST120kV protocol had significantly higher CNR values and lower background noise than the TF100kV protocol for imaging abdominal organs. Leyendecker et al. [25] similarly compared objective metrics of the TF100kV protocol with their standard protocol for contrast-enhanced abdominopelvic CT imaging but found that CNR and background noise within anatomical structures were comparable. Their contrasting findings may be due to differences between standard protocols and CT scanner performance, where standard protocol images were acquired using automatic tube voltage selection, and all examinations were acquired on a third-generation dual-source CT system [25]. In contrast, our standard protocol images were acquired at a spectrum of 120 kV in a single-energy setting, using a single-source CT system. These differences may have contributed to the lower CNR and higher background noise of the TF100kV protocol in our study. Despite lower CNR values of the abdominal organs and higher background noise, the TF100kV images retained good diagnostic performance for postoperative follow-up and chemotherapy examinations in CCPs and were considered usable in clinical practice. The CTDIvol and DLP of the TF100kV CAP CT images were substantially lower than those of the national diagnostic reference levels of the United States and Japan [33,34]. Although developing techniques to improve image quality of TF-based spectral shaping CT images is important, given our commitment to the ALARA principle, objective image quality metrics may not be crucial for determining the optimal imaging protocol, as long as diagnostic ability can be ensured.

Our study had several limitations that need to be considered. First, this study could not rigorously assess the changes in the number and size of metastases in each organ of patients during chemotherapy between the acquired images of each protocol. We believed that the TF100kV protocol maintained comparable diagnostic performance to the ST120kV protocol, regardless of the difference in image quality; however, the diagnostic performance might also be affected by the number and size of metastases in each organ. In patients during chemotherapy, the number and size of lesions in each site might have slightly progressed as of the TF100kV scans. As the number and size of lesions increased, regardless of the effect of image quality, there was a risk that the rater’s ability to detect lesions would increase (became more sensitive). On the other hand, if chemotherapy slightly reduced the number and size of lesions compared to the ST120kV image, detectability performance might be reduced (increased false-negative cases). Since CT involves radiation exposure, it is not desirable to perform unnecessary multiple scans. However, to evaluate only the effect of the difference in image quality for the diagnostic performance of the subjective assessments, the ST120kV scan and TF100kV scan would have to be performed simultaneously to eliminate the impact of lesion number and size. Second, the study was conducted in a single center retrospective design, and the cohort size was relatively small. Third, the presence of most listed metastases was defined by radiology reports and clinical data and not by pathology. To prevent underdiagnosis of metastases as much as possible when determining the standard reference on each CT protocol, the data acquisition timing in this study was more than a year after TF100kV scans. Therefore, new metastases appeared through the clinical course after TF100kV scans in some cases, but those lesions were not depicted as the mass in the TF100kV image. They were determined as the absence of metastasis as of the CT scans. However, there was a still risk that the lesion was already pathologically present at the TF100kV scans. In clinical practice, it is often challenging to obtain pathological confirmation for diagnosing metastasis in all CCPs during postoperative follow-up or chemotherapy, and CT imaging is regarded as a usual diagnostic method for diagnosing metastasis. Further validation studies in larger populations are needed to evaluate the detectability of metastatic lesions.

## 5. Conclusions

In conclusion, we found that TF100kV CAP CT substantially reduced radiation dose by approximately 89% compared with ST120kV CAP CT while maintaining a good diagnostic performance in CCPs. The TF-based spectral shaping technique may alleviate concerns of excessive medical radiation exposure of CCPs who require routine surveillance and response evaluation using CT.

## Figures and Tables

**Table 1:** CT acquisition scanning parameters for both standard and spectral filtration protocols.

CT Parameters	ST120kV Protocol	TF100kV Protocol
Tin-filter ^a^	off	on
kV	120 kV	100 kV
Quality reference mAs ^b^	320 mAs	600 mAs
Rotation time	0.5 s	0.5 s
Beam collimation	128 ch × 0.6 mm	128 ch× 0.6 mm
Beam pitch	0.6	0.5
Kernel	Bf37 (Bl57 for lung area)	Bf37 (Bl57 for lung area)
Reconstruction technique	IR strength 2 for parenchymal images	IR strength 4 for parenchymal images
	FBP for lung images	FBP for lung images

ST120kV, standard 120 kV; TF100kV, tin-filtered 100 kV; IR, iterative reconstruction (ADMIRE; Advanced Modeled Iterative Reconstruction); FBP, filtered back-projection technique. ^a^—Thickness of tin-filter was 0.6 mm. ^b^—Automatic tube current modulation (CARE Dose 4D) was turned on.

**Table 2:** Patient and clinicopathological characteristics of the 44 eligible patients.

Variables	*n* (%)
Age (y) ^a^	65.1 (38–88)
Gender	
Male	30 (68)
Female	14 (32)
Total number of colorectal cancers ^b^	45
Cecum/Appendix	3
Ascending colon	4
Transverse colon	3
Descending colon	1
Sigmoid colon	12
Rectum	22
Stage	
Ⅰ, Ⅱ	10 (23)
Ⅲ, Ⅳ	34 (77)
Disease-free and during follow-up	26 (59)
During chemotherapy	18 (41)
Total number of metastatic site ^c^	32, 30
Lung metastasis ^c^	9, 9
Liver metastasis ^c^	9, 8
Lymph node metastasis ^c^	6, 5
Peritoneal dissemination ^c^	5, 5
Bone metastasis ^c^	3, 3
Time from ST120kV images to TF100kV images (d) ^a^	156 (65–240)

ST120kV, standard 120 kV; TF100kV, tin-filtered 100 kV. ^a^ Mean (range). ^b^ One patient had two separate synchronous cancers (transverse and sigmoid colon). ^c^ Data are the number of patients as of the ST120kV and TF100kV scans.

**Table 3:** Radiation metrics of each protocol.

	ST120kV Protocol	TF100kV Protocol	Reduction Rate Using TF100kV Protocol (%)	*p* Value
CTDI_vol_ (mGy) ^a^	14.4 ± 2.50	1.60 ± 0.31	88.9	<0.0001
DLP (mGy·cm) ^b^	996.7 (886.2–1144.3)	107.1 (95.9–125.5)	89.3	<0.0001
Effective dose (mSv) ^b^	17.9 (16.0–20.6)	1.93 (1.73–2.26)	89.2	<0.0001
SSDE (mGy) ^a^	20.8 ± 1.90	2.29 ± 0.24	89.0	<0.0001

ST120kV, standard 120 kV; TF100kV, tin-filtered 100 kV. ^a^ Data are presented as means ± standard deviations. ^b^ Data are presented as median (interquartile range).

**Table 4:** Objective assessment of image quality.

	ST120kV Protocol	TF100kV Protocol	*p* Value
CNR of liver	2.76 (1.38–4.03)	2.13 (1.42–2.73)	<0.0001
CNR of abdominal aorta	5.81 (4.76–7.52)	4.08 (3.39–5.02)	<0.0001
FOM of liver (mSv^−1^)	0.36 (0.09–0.93)	2.24 (0.89–4.07)	<0.0001
FOM of abdominal aorta (mSv^−1^)	1.81 (1.19–3.16)	8.62 (5.98–14.1)	<0.0001
Background noise	6.04 (5.45–6.93)	8.36 (7.61–8.86)	<0.0001

ST120kV, standard 120 kV; TF100kV, tin-filtered 100 kV. Data are presented as median (interquartile range).

**Table 5:** Subjective assessment of diagnostic performance with Kappa agreement.

	Diagnostic Performance								Interreader Agreement	
	ST120kV				TF100kV				ST120kV	TF100kV
Diagnostic of	SN (%)	SP (%)	AC (%)	AUC ^a^	SN (%)	SP (%)	AC (%)	AUC ^a^	Kappa	Kappa
Lung metastasis	89 [8/9]	100 [35/35]	98 [43/44]	0.94	100 [9/9]	100 [35/35]	100 [44/44]	1.0	0.59	0.66
Liver metastasis	67 [6/9]	100 [35/35]	93 [41/44]	0.83	75 [6/8]	100 [36/36]	96 [42/44]	0.88	0.72	0.44
Lymph node metastasis	83 [5/6]	97 [37/38]	96 [42/44]	0.90	40 [2/5]	100 [39/39]	93 [41/44]	0.70	0.39	0.33
Peritoneal dissemination	60 [3/5]	95 [37/39]	91 [40/44]	0.77	60 [3/5]	97 [38/39]	93 [41/44]	0.79	0.17	0.29
Bone metastasis	67 [2/3]	100 [41/41]	98 [43/44]	0.83	67 [2/3]	100 [41/41]	98 [43/44]	0.83	0.23	0.37

ST120kV, standard 120 kV; TF100kV, tin-filtered 100 kV; SN, sensitivity; SP, specificity; AC, accuracy; AUC, area under the curve. ^a^—AUCs were calculated using the receiver operating characteristic curve.

**Table 6:** Diagnostic reliability of tin-filtered 100 kV image quality.

	Reader 1	Reader 2
Lung metastasis	4.00 (4.00–4.00)	4.00 (4.00–4.00)
Liver metastasis	3.68 (3.00–4.00)	3.32 (3.00–4.00)
Lymph node metastasis	3.89 (3.00–4.00)	3.91 (3.00–4.00)
Peritoneal dissemination	3.86 (3.00–4.00)	3.91 (3.00–4.00)
Bone metastasis	4.00 (4.00–4.00)	4.00 (4.00–4.00)

Data are presented as mean (range).

## Data Availability

Not applicable.
